# Investigation into limiting dilution and tick transmissibility phenotypes associated with attenuation of the S24 vaccine strain

**DOI:** 10.1186/s13071-019-3678-2

**Published:** 2019-08-27

**Authors:** Ben J. Mans, Ronel Pienaar, P. Christo Troskie, Michael P. Combrink

**Affiliations:** 1 Epidemiology, Parasites and Vectors, ARC-Onderstepoort Veterinary Institute, Private Bag X05, Onderstepoort, 0110 South Africa; 2 Department of Life and Consumer Sciences, University of South Africa, Florida, South Africa

**Keywords:** *Babesia bovis*, Vaccine, Tick transmission, Limiting dilution, Sequestration, Genetic recombination

## Abstract

**Background:**

*Babesia bovis* is the causal agent of Asiatic redwater, transmitted by the pandemic tick *Rhipicephalus* (*Boophilus*) *microplus*. Disease control may target the tick vector using acaricides or anti-tick vaccines, or the parasite using chemoprophylaxis or anti-parasite vaccines. Current anti-parasite vaccines comprise live blood vaccines using attenuated *B. bovis* strains. Attenuation is attained by rapid passage that may result in different phenotypes such as reduced virulence, non-transmissibility by the tick vector, inability to sequester in the host (lack of limiting dilution) and limited genetic diversity. Attenuation and phenotypes may be linked to selection of subpopulations during rapid passage. The South African *B. bovis* S24 vaccine strain comprise a subpopulation that present low virulence, non-transmissibility, lack of limiting dilution phenotype and the presence of a single A558 Bv80 allele. The S24 strain could be co-transmitted with a field strain (05-100) suggesting sexual recombination. The present study investigated the change in phenotype for the S24 vaccine strain during rapid passage and co-transmission.

**Methods:**

Vaccine phenotype change during passage as well as co-transmissibility was monitored using Bv80 allele specific PCR, limiting dilution and Illumina-based genome sequencing.

**Results:**

The S24 population could not be rescued from the S16 passage as previously attained suggesting that selection of the S24 vaccine strain was a serendipitous and stochastic event. Passage from S16 to S24 also resulted in loss of the limiting dilution phenotype. Genome sequencing indicated sexual recombination during co-transmission with the 05-100 field strain. Analysis of the recombinant strain indicate that VESA1, smORF and SBP2 family members are present and may be responsible for the limiting dilution phenotypes, while various regions may also be responsible for the tick transmission phenotype.

**Conclusions:**

The molecular basis for tick transmission and limiting dilution phenotypes may be defined in future using selection based on these traits in combination with sexual recombination.
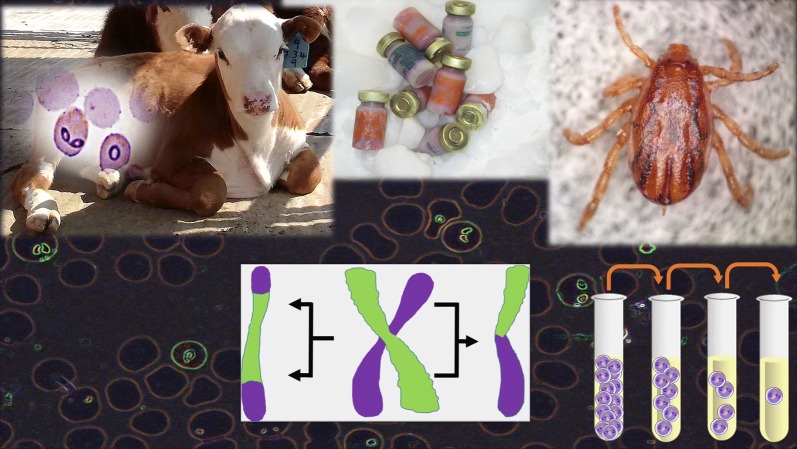

## Background

Asiatic redwater is a global important disease in cattle caused by *Babesia bovis* and transmitted by members of the *Rhipicephalus* (*Boophilus*) *microplus* complex [1]. The *Rhipicephalus* (*B.*) *microplus* complex is found on all major continents including North America, South America, Africa, Asia and Australia [2]. Members of the complex include *R.* (*B.*) *australis*, *R.* (*B.*) *annulatus*, *R.* (*B.*) *microplus* (*sensu stricto*) as well as several genotypes not yet well described [3, 4]. The vector and the disease were historically confined to savanna regions of wooded grasslands, due to restrictions of humidity and temperature [5–8]. In South Africa, the historical distribution of *R.* (*B.*) *microplus* ranged from grassland biomes of the Eastern Cape, southern regions of KwaZulu-Natal and north-eastern regions of Limpopo and North-West Provinces, with scattered localities in Mpumalanga [8]. However, more recently its distribution range was extended to the coastal region of the Eastern Cape and into some areas of the Northern and Western Cape, as well as the Free State [9, 10]. The South African tick strains are genetically related to *R.* (*B.*) *microplus* (*sensu stricto*) as found in South America [3, 11, 12].

Control of Asiatic red water may be accomplished through tick control using acaricides or the Bm86 antigen anti-tick vaccines (GAVAC or TickGard) [13, 14]. However, in many countries significant acaricide resistance has been observed [14], while the Bm86 vaccines have served a limited commercial niche to date [13]. The alternative control strategy is targeting of the *B. bovis* parasite through prophylaxis or vaccination [15, 16]. Prophylaxis comprises treatment of clinically sick animals with diminazene aceturate. The drawback of this lies in the timeous identification of clinically sick animals, since severe clinical symptoms may only present a few days before the onset of acute illness and death. This has led to the practice of block treatment of herds when a redwater outbreak is suspected, or when treating vaccinated animals using the infection and treatment method [16]. Vaccination generally comprises live vaccines produced in animals or cell culture [15]. Although a promising alternative, development of subunit vaccines has not proved to be successful yet [17].

Attenuation of live vaccines to reduce virulence, by repeated syringe passage through splenectomized animals has been important in the development of effective vaccines against redwater [18]. Passage may also result in selection of additional phenotypes such as inability of transmittance by ticks or loss of efficacy [19–21]. In some cases, reversion to virulence has been observed, even in non-tick transmissible strains [18, 20]. These phenotypic transformations may be due to parasite subpopulations with varying virulence or tick-transmissibility, selected during serial blood passage, or even genetic changes in clonal parasite populations [19]. Since all vaccine stocks derive from regional specific parasite populations that was selected through serial passage bottlenecks [15], it may be expected that each geographical vaccine stock may present unique genetic makeups and phenotypic properties obtained *via* a stochastic process. Dissection of the genetic makeup of these passaged vaccine stocks may therefore illuminate characters responsible for various phenotypes, or shared in efficacious vaccines.

In South Africa, a virulent *B. bovis* S strain was rapidly syringe passaged ten times in splenectomized calves to yield the S10 strain [22, 23]. The S10 strain was less virulent than the original S strain and was used from 1978 as the S11 passage. However, severe reactions in calves still occurred, leading to an additional thirteen rapid syringe passages through splenectomized calves to obtain the S23 strain [21]. This strain retained efficacy, but was less virulent than S11 and has been used in the commercial vaccine as S24 since 1981 [21]. The S24 vaccine strain is non-transmissible in ticks, limiting its potential to revert to virulence or spreading disease through transmission [21, 24]. More recently, its non-transmissibility was confirmed, but it was shown that co-transmission is possible when an animal is infected with additional *B. bovis* strains, such as the 05-100 field strain [21]. This has also been observed for Australian *B. bovis* vaccine strains that was rapid passaged for 20–30 times, that still contained transmissible and non-transmissible clones [19, 20]. This suggested that transmissible subpopulations may support non-transmissible subpopulations by providing transmission factors either exogenously, or via sexual recombination. It also suggested that the South African S24 vaccine strain is probably a clonal strain that lacks any transmissible subpopulations [21].

Dissection of the genetic makeup of the S24 vaccine strain using the Bv80 gene showed that the genetic composition of the vaccine changed over time from the S11 to the final S24 strain [25]. This was related to a change in *B. bovis* population complexity and makeup during the rapid syringe passages in splenectomized cattle. The S11 strain possessed both A and B Bv80 alleles of A645 and B585, respectively, while the S24 strain only possessed a single A allele of A558 (the prefix A or B denote the A or B allele of the Bv80 gene, while the size of the band is indicated in base pairs) [25]. This previous study indicated a marked change in allele populations between passage S16 (S11 profile) and passage S18 (S24 profile), indicating a changeover in populations within one or two passages. The present study investigated this *B. bovis* population change in more depth to determine whether selection of the S24 allele was a random event, or whether conditions during passages affected the dramatic change from dominant A645 and B585 populations to the single A558 genotype observed in S24. In addition, genome sequencing was used to show that sexual recombination occurs during co-transmission of the S24 and 05-100 field strain.

## Methods

### Animals

Cattle used for vaccine serial passage (*n* = 2; 8-month-old), transmission (*n* = 14; ≥ 5-year-old) and cloning (*n* = 8; 9- to 15-month-old) studies were all fully susceptible splenectomized Herefords, reared and kept from birth under tick free quarantine conditions at the ARC-OVR.

### Tick vector

A colony of uninfected *R.* (*B.*) *microplus* larvae maintained at the ARC-OVR were used for the experimental infection of adult females with *B. bovis* and subsequent transmission feeding of the larval progeny on susceptible splenectomized animals. Replete females that dropped from the uninfected control and infected animals were kept at 24 °C and 75% relative humidity with a circadian day and night cycle of 12 h. The hemolymph of these ticks was examined for *B. bovis* kinetes from which the infection rate per tick batch were determined (number of infected ticks divided by *n* = 20 ticks examined per batch, expressed as %). For the transmission attempts, each bovine received the progeny of 1g (± 20,000) larvae, pooled from 15 females that were distributed on the back line of cattle allowing for natural unrestricted movement on the animal’s body to the preferred sites of attachment for this species.

### Passaging of vaccine strains

The *B. bovis* S16 vaccine stabilate used for intravenous serial passage was from the 1981 deep frozen stock containing ± 2.7 × 10^8^ parasites/ampoule at time of freezing. To distinguish between the numbering of original unfrozen serial passaging (S12 to S22) and revival passaging with frozen S16 material, the suffix ‟.2” was added to the generation number. Infected S17.2 blood was collected for cloning by limiting dilution and PCR study in ethylenediaminetetraacetic acid (EDTA), for serial passaging in acid-citrate-dextrose (ACD) anticoagulant (2.16 × 10^8^ parasites/ml) and for deep freezing using dimethyl sulfoxide (DMSO) as cryoprotectant. Blood for serial passaging was kept for 7 days at 4 °C (simulating the original procedure followed during unfrozen serial passaging from S11 to S22), where after 5 ml was administered intravenously to a bovine and from which at the peak of the reaction S18.2 infected blood were collected for PCR.

### Cloning by limiting dilution

For cloning by limiting dilution, fresh *B. bovis* S17.2 parasite infected blood was collected at the peak of the animal’s reaction. The number of parasites per ml blood was determined (percentage of infected red blood cells multiplied by the number of red blood cells per ml of blood) and uninfected donor blood was used to dilute the blood to theoretically contain 3 parasites in 4 ml (Table 2) [21]. Eight cattle were each inoculated intravenously with 4 ml of the dilution within five hours after collection of the infected blood from the donor animal. Animals were monitored 30 days for infection. Blood for PCR tests and cryopreservation of clonal lines was collected in EDTA during the acute stage of animal reactions, or after 30 days for non-reactors. One of the clonal lines obtained, 9526-17.2-cl (genotypic similar to the Bv80 A558 population found in the S24 vaccine) were selected for further tick infection and transmission study (Table 1). Clonal line designations refer to the animal used indicated by its unique number (9526), the origin of the isolate (S17.2) and its clonal nature (cl).

**Table 1:** *Rhipicephalus* (*B.*) *microplus* transmission of the *B. bovis* S17.2 and S18.2 vaccine strains, and the S17.2 clonal line parasite population

Group	*B. bovis* strains	*B. bovis* Bv80 genotype classification according to alleles and base pairs	No. of cattle used for tick feeding	Parasitaemia during dropping of engorged females (%)	Percentage female ticks hemolymph positive for kinetes out of 20 examined	No. of cattle each infested with ± 20,000 larvae	*B. bovis* genotype(s) detected following transmission
1	S17.2 (S16)^a^	A558; A627; A645; B585	2	0.20–1.5	95	1	A558; A627; A645; B585
				0.08–1.0	100	1	A558; A627; A645; B585
2	S18.2 (S17.2)^a^	A645; B585	2	0.60–1.7	100	1	A645; B585
				0.20–1.2	50	1	A645; B585
3	Not infected	–	1	–	0	1	No transmission
4	9626-S17.2-cl (originating from 3 parasites of S17.2)^a^	A558	2	0.10–1.0; 0.09–0.8	0; 0	1; 1	No transmission

^a^Stabilate used for infection

### Strains and clonal line used for tick pick-up

*Babesia bovis* vaccine used for tick infection by *R.* (*B.*) *microplus* were from the frozen stabilates of S16 and S17.2 and were administered intravenously to 2 individual animals each at doses containing 2.5 × 10^7^ and 5 × 10^7^ parasites, respectively. The clonal line 9526-17.2-cl used was from frozen stock and administered intravenously to 2 animals at 5 × 10^7^ parasites per single dose (Table 1). Animals were infected at such a time as to ensure that the presence of engorging adult females coincided with the parasitaemic period for the specific inoculum. Once replete, dropped off ticks were collected, sorted according to inoculum and the larvae used for transmission study.

### Monitoring infections

Cattle were monitored daily (30 days) for rectal temperatures, packed cell volumes (PCV) and Giemsa stained blood smears. Infectivity in cattle was determined by demonstrating the *B. bovis* parasites in stained blood smears. Tick infection rate, expressed as percentage positive, was determined by demonstrating *B. bovis* kinetes in hemolymph smears prepared from 20 females that were randomly selected on day 10 of the oviposition period. EDTA-blood was collected during the clinical reaction as well as weekly for the 30-day period for analysis using PCR.

### PCR and analysis of genotypes

EDTA-blood (200 μl) was extracted using the MagNAPure LC (Roche) and DNA eluted into 100 μl elution buffer (50 mM Tris-HCl, pH 8.0) as described [26]. All samples were confirmed negative for *B. bigemina* by PCR amplification using primers specific for B. *bigemina* (BBIA: 5′-CAT CTA ATT TCT CTC CAT ACC CCT CC-3′; BBIB: 5′-CCT CGG CTT CAA CTC TGA TGC CAA AG-3′) [27]. Genotypes were analyzed using the Bv80 allele specific primers for allele A (BbAF: 5′-GTA GTG GAG CCC ACT GAA GAG CCG GCT GGC-3′; BbAR: 5′-GCC ACA TTT GGG TAC AAG ATT ACA AGA AGC-3′) and allele B (BbBF: 5′-GAG CAG CCA GTT GCT GAA GAA CCA TCT GAT-3′; BbBR: 5′-TTC ACC TTT GCG ACC ACC GTA ACA AGG TCT-3′) [25]. Amplification was performed using a touch-down procedure that included an initial denaturation at 95 °C (2 min) followed by denaturation at 95 °C (30 s), annealing at 65–55 °C (30 s), extension at 72 °C (2 min) for 10 cycles, followed by 40 cycles using an annealing temperature of 55 °C. The High Resolution Cartridge of the QIAxcell system (Qiagen, Hilden, Germany) [28] was used to analyze the samples. As standard, the 100 bp O’Gene Ruler ladder was used (Fermentas, Vilnius, Lithuania) and the peaks integrated using the QIAxcell software.

### Isolates selected for genome sequencing

Isolates selected for genome sequencing included three clones obtained from the S17.2 limiting dilution, namely 9622-S17.2cl (A558), 9623-S17.2cl (A535) and 9626-S17.cl (A558). In addition, the S24 vaccine isolate was sequenced (9512-S24) and the 05-100 field strain (9547-05-100). The latter strain possesses a single B615 allele for Bv80 and enabled co-transmission of the S24 vaccine strain previously [21, 25]. Three clones obtained by limiting dilution of the co-transmitted S24 and 05-100 strains, namely 9480-S24×05-100, 9574-S24×05-100 and 9563-S24×05-100 were also sequenced to detect possible sexual recombination between S24 and 05-100.

### Genome sequencing using Illumina HiSeq

Blood was sampled (100 ml), from infected animals into EDTA tubes and red blood cells washed five times by pelleting at 845×*g* for 10 min, removing the supernatant and buffy coat layer and resuspending in an equal volume of phosphate buffered saline (PBS). The remaining red blood cells were resuspended in an equal volume of water and passed through a cellulose column to remove bovine lymphocytes [29]. The flow through was used for genomic DNA extraction using the Roche MagNA Pure system as described [30]. Genomic DNA was submitted to the Biotechnology Platform Next Generation Sequencing Service of the Agricultural Research Council (Onderstepoort, South Africa). Samples were processed using Nextera DNA preparation kits (Illumina, San Diego, CA, USA) and sequenced using the Illumina HiSeq 2500 sequencer.

Raw paired Illumina reads were quality trimmed using the BBDuk (Bestus Bioinformatics Decontamination Using Kmers) program in the BBTools (Bestus Bioinformatics Tools) suite (https://jgi.doe.gov/data-and-tools/bbtools/). For trimming, a single base pair was removed from each read and then Nextera and Illumina adapters were removed using specified parameters (ktrim = r, k = 21, mink = 11, hdist = 2, tpe, tbo) using the BBDukF command. PhiX contamination was then removed using the same command with specified parameters (k = 31, hdist = 1). Paired reads were quality trimmed to Q20 using specified parameters (qtrim = r, trimq = 20), reads with quality below 20 were discarded (maq = 20) and reads below 50 bp were removed (qtrim = r, trimq = 10, minlen = 50). Reads were then normalized using the BBNorm program, to either an average depth of 100× (discarding reads with coverage below 20) or an average depth of 50× (discarding reads with coverage below 10), depending on the amount of sequence data generated initially. Quality trimmed and normalized paired sequence datasets were then used for assembly. Assemblies were performed using the CLC Genomics Workbench v 11 software (Qiagen). Reads were *de novo* assembled using standard assembly parameters: mismatch cost-2, insertion cost-3, deletion cost-3, length fraction-0.5, similarity-0.9, minimum contig length-200, automatic bubble size and a variable word size (kmers). For each sample a range of assemblies with different kmers (64, 60, 55, 50, 45, 40, 35, 30, 25, 20 and 15) were performed. Assembled contigs were extracted, compared to the reference genome using BLASTN analysis and chromosome specific contigs mapped to the reference genome [31], to produce a final assembled genome scaffold for each sample from which consensus genome sequences were derived. Parameters from the different assemblies and coverage may be accessed in Additional file 1: Table S1, Additional file 2: Table S2 and Additional file 3: Table S3.

### Genome comparisons

To compare genomes, they were partitioned into 1000 bp fragments and compared to other assembled genomes using BLASTN analysis to obtain both pairwise identities and size of the longest aligned hit [32]. This was plotted across the genome length to assess potential recombination. Assembled genomes were also aligned using Mauve with default parameters [33]. Gaps were removed from the alignment using GBlocks [34], to enable manual inspection for recombination cross-over and phylogenetic analysis.

### Phylogenetic analysis

To determine relationships of the various sequenced clonal lines, the aligned genomes were analyzed by Maximum Likelihood analysis using IQ-Tree v1.5.2 [35]. Optimal substitution models were estimated for each aligned chromosome partition: Chr1a + b (TVM + I + G4), Chr2 (TVM + I + G4), Chr3 (TVM + I), Chr4a, Chr4b (TVM + I + G4). An edge-proportional partition model with proportional branch lengths (-spp) was used to allow each partition its own specific rate to accommodate different evolutionary rates between partitions. Nodal support was estimated using ultrafast bootstrap (*n* = 100,000) and the 50% consensus tree was reported. The number of sites used in the analysis was 6,629,566 sites.

### Variant detection within and between genome datasets

Genetic diversity within and between genome datasets were investigated by mapping datasets to the respective consensus genomes obtained for the various clonal lines using CLC Genomics Workbench v 11 software (Qiagen). Parameters for mapping were match score 1, mismatch cost 2, insertion cost 3, deletion cost 3, length fraction 0.5 and similarity fraction 0.9. Parameters for basic variant detection were ploidy 1, exclusion of positions with coverage 2× above the average genome coverage and exclusion of broken pairs. Positions were considered with a minimum coverage of 10, minimum read count of 10 and 50% frequency.

## Results

### Transmission of S16 and S17.2 vaccine strains

**Fig. 1:**
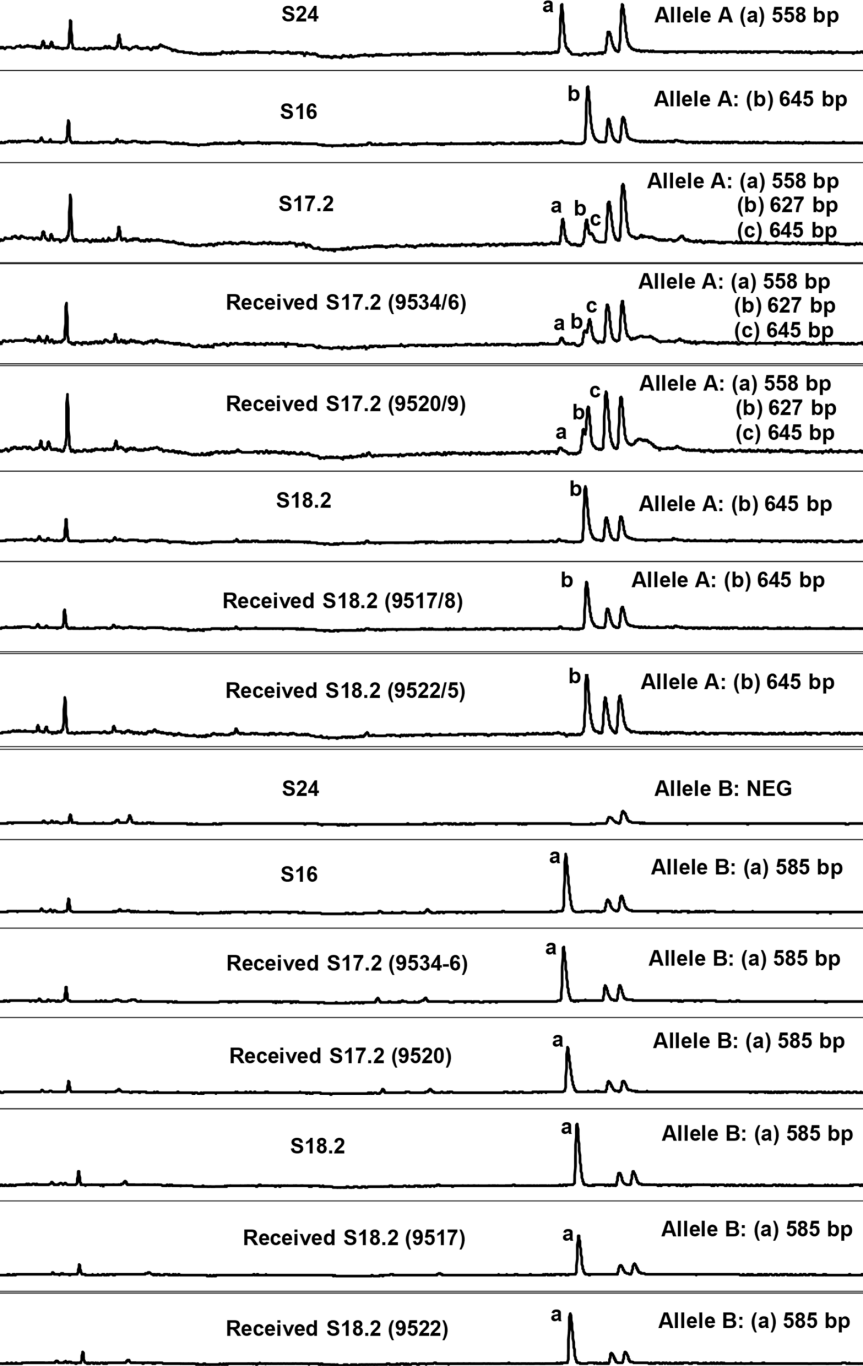
Analysis of the Bv80 alleles for various passages. Indicated are the sizes found for either allele A or allele B as well as the identity of the isolates or clonal lines

The genetic makeup of the S16 passage was similar to previous work [25], indicating a major A645 allele and a major B585 allele (Fig. 1). Infection by needle challenge to produce the S17.2 strain resulted in the appearance of the A558, A627 and A645 genotypes, as well as the B585 genotype (Fig. 1). Tick pickup (Group 1) resulted in a high infection rate (95% and 100%, *n* = 20 each) and tick transmission recovered all alleles in two separate transmission events (Fig. 1, Table 1). Admittedly, the A558 allele was almost non-detectable for either transmission. A single passage from S17.2 that yielded the S18.2 strain resulted in loss of both A558 and A627 alleles, with only the A645 and B585 alleles remaining. Tick pickup (Group 2) resulted in infections rates of 100% and 50% (*n* = 20 each) with transmission of both alleles (Fig. 1, Table 1). Ticks (*n* = 20) collected from the uninfected control animal (Group 3) showed no kinetes in the haemolymph after feeding and no parasites were demonstrated in the animal used for transmission feeding (Table 1).

### Clonal lines obtained from the S17.2 vaccine strain

Since the A558 allele was lost during passage of the S17.2 strain, limiting dilution of this latter strain was attempted to recover this allele. Infection of splenectomized cattle (*n* = 8) with limiting dilution inoculums (theoretically containing 3 parasites), yielded four A558 clones similar to that found in the S24 vaccine and two clones of A535 not previously observed (Table 2).

**Table 2:** Cloning by limiting dilution of the *B. bovis* S17.2 vaccine strain for group 1 (Table 1)

Bv80 genotype classification according to alleles and base pairs	Theoretical no. of *B. bovis* parasites in 4 ml inoculum	No. of splenectomized cattle inoculated	No. of single genotype clonal lines obtained by limiting dilution	
			A535	A558
A558; A627	3	8	2 (25)^a^	4 (50)^a^
A645; B585				

^a^Percentage of cattle that became infected in parentheses

### Transmission of S17.2-cl

A single clone, 9626-S17.2-cl (A558) of the six *B. bovis* clonal lines obtained by limiting dilution of S17.2 were selected for further transmission studies (Table 1). This parasite clonal line (Group 4) failed to infect ticks in 2 feeding attempts and could not be transmitted to 2 susceptible bovines. No kinetes were demonstrated in the hemolymph of both tick batches (*n* = 20) used for infection.

### Genomic comparisons of S17.2-cl, S24 and crosses with the 05-100 field strain

**Fig. 2:**
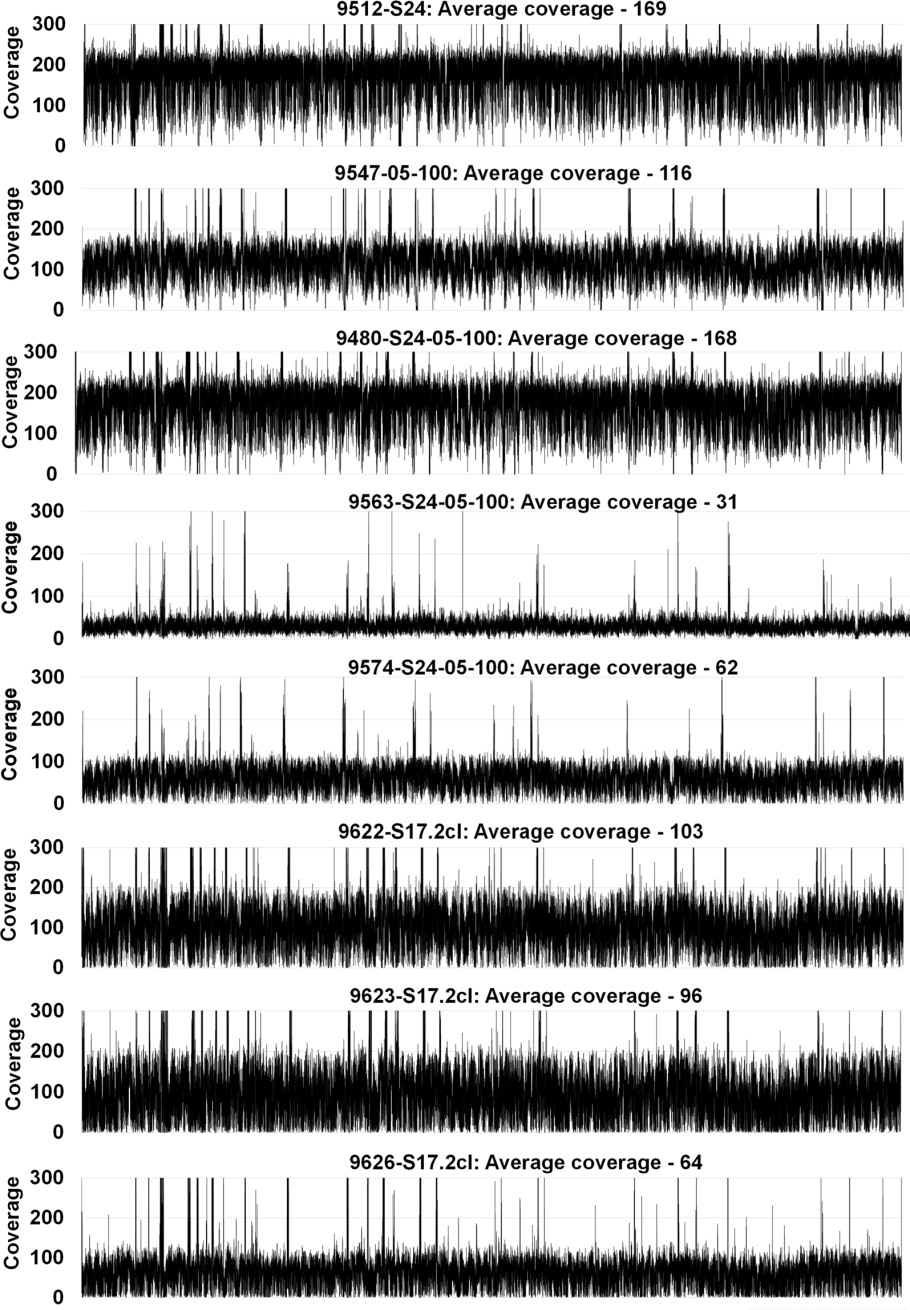
Coverage of the various assembled *B. bovis* genomes for the major chromosomes (Chr1a, Chr1b, Chr2, Chr3, Chr4a, Chr4b) concatenated together. Included is the overall average coverage

Previous attempts to recover the S24 vaccine strain by limiting dilution failed on numerous occasions [21]. The question was therefore raised regarding the relationship of the A558 genotypes from the S16 and the S24 passages, since they exhibit different limiting dilution phenotypes (ability to proliferate after limiting dilution *vs* the inability to proliferate), even if both were non-transmissible and exhibit the same A558 genotype. To address this, the genomes of three S17.2-cl clones (9622-S17.2cl [A558], 9626-S17.2cl [A558]; 9623-S17.2cl [A535]) as well as the S24 vaccine strain were sequenced. Genomes were *de novo* assembled and contigs scaffolded onto the reference genome resulting in assemblies that represented > 90% of the major chromosomes of the original Texas T2Bo *B. bovis* genome (chromosomes 1a, 1b, 2, 3, 4a and 4b) [31]. Average coverage ranged from 31 to 169 (Fig. 2, Additional files 1, 2, 3: Tables S1, S2, S3) and was even across the major chromosomes for each assembled genome, suggesting that all regions are well represented in the final assemblies (Fig. 2).

**Fig. 3:**
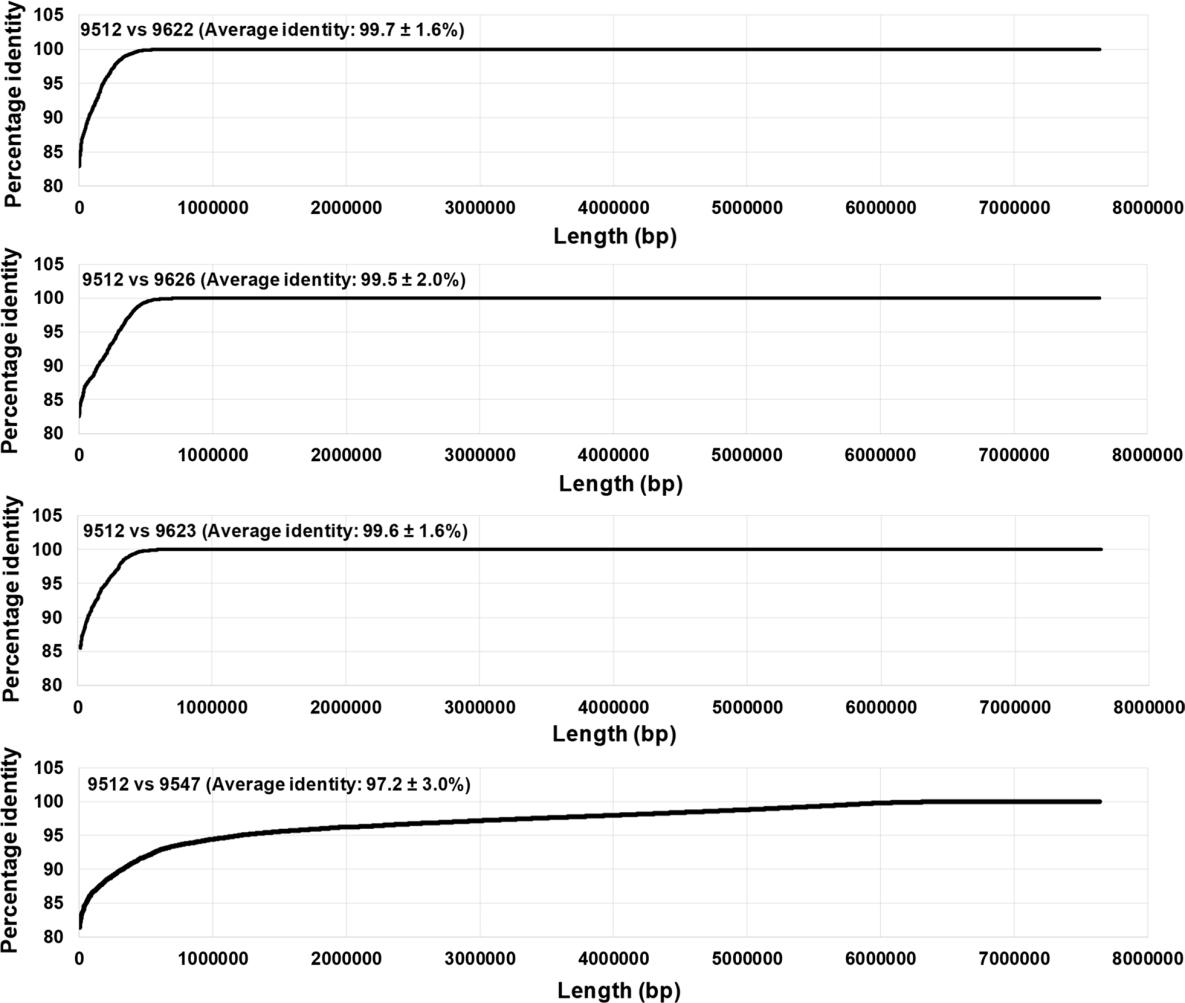
Comparison of assembled genomes from the S17.2-cl clonal lines 9622-S17.2-cl, 9623-S17.2-cl, 9626-S17.2-cl as well as the field isolate 9547-05-100 with 9512-S24. Indicated are pairwise sequence comparisons of 1000 bp fragments from 9512-S24 against various genomes. Percentage identity values for these fragments were sorted from low to high percentage and plotted against the combined length of the genome. The average pairwise identity of all fragments (*n* = 7640 ± SD) are also indicated

**Fig. 4:**
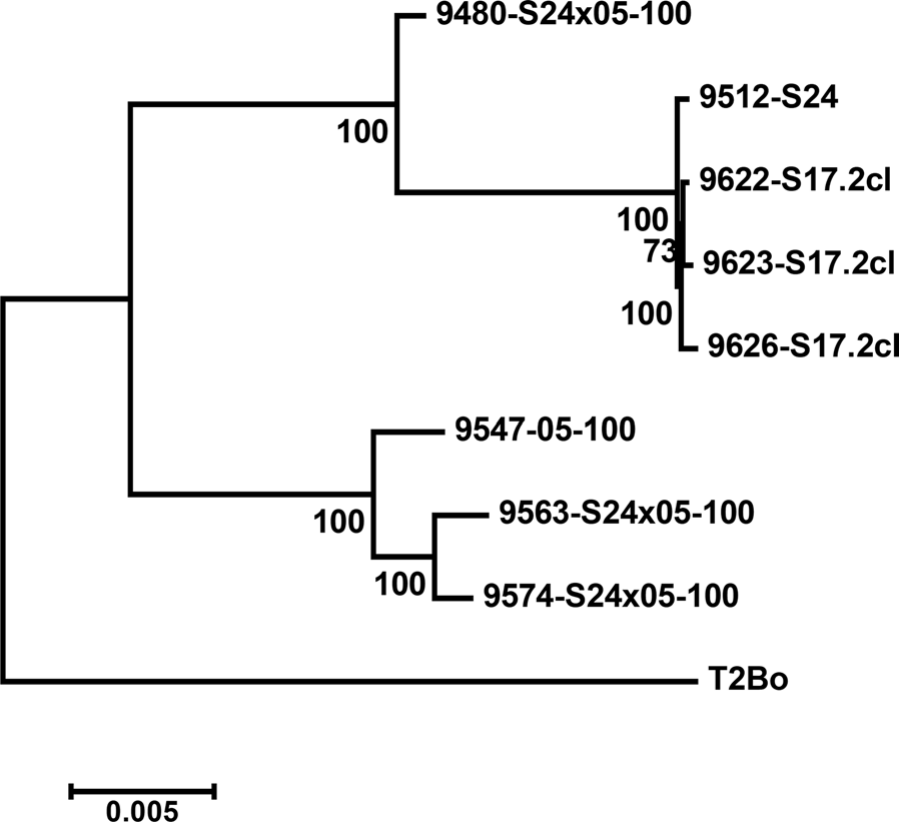
Maximum Likelihood analysis of the various *Babesia* genomes. Total sites used were 6,629,566 sites with all gaps removed. Bootstrap support (*n* = 100,000) are indicated and the 50% consensus tree presented. The tree was rooted with the T2Bo genome

Sequence comparison of 9622-S17.2-cl (A558), 9626-S17.2-cl (A558) and 9512-S24 (A558) over a combined length of 6.5-6.9 Mbp (alignment of Chr1a, Chr1b, Chr2, Chr3, Chr4a and Chr4b, with gaps removed), indicated 100% identity (Fig. 3). This combined length represents 80–85% of the original T2Bo genome and indicate that the A558 bp genotypes from the S16 and S24 passages derive from the same parasite population. While several truncated or fragmented genes exist between the genomes that may explain the limiting dilution phenotypic differences, these may be due to assembly artefacts and need further investigation. Interestingly, clone 9623-S17.2-cl (A535) also show 100% identity to S24 (A558) over a combined length of 6.7 Mbp that represents 83% of the original *B. bovis* genome. It suggests that A535 also derive from the S24 ancestral population and that genetic changes can occur within the time course of several passages. This is supported by phylogenetic analysis based on whole genome alignment of 6,629,566 sites that indicated that 9622-17.2-cl, 9623-17.2-cl, 9626-17.2-cl and 9512-S24 group in a well-supported clade with small intra-clade genetic distances (Fig. 4), suggesting very close genetic relationships as may be expected if these derived from the same ancestral population. Conversely, comparison to field strain 9547-05-100 (B615), previously used in a co-transmission study [21], indicated that the average identity was ~97% compared to S24 (A558), while 100% identity was only observed for a combined length of 1.3 Mbp, comprising 16% of the original T2Bo genome length distributed homogenously throughout the genome. This would suggest a closer genetic relationship between the vaccine derived clones compared to the field strain. This is supported by phylogenetic analysis that indicated that the vaccine and field strains group in different clades (Fig. 4).

### Tick transmissibility and sexual recombination

**Fig. 5:**
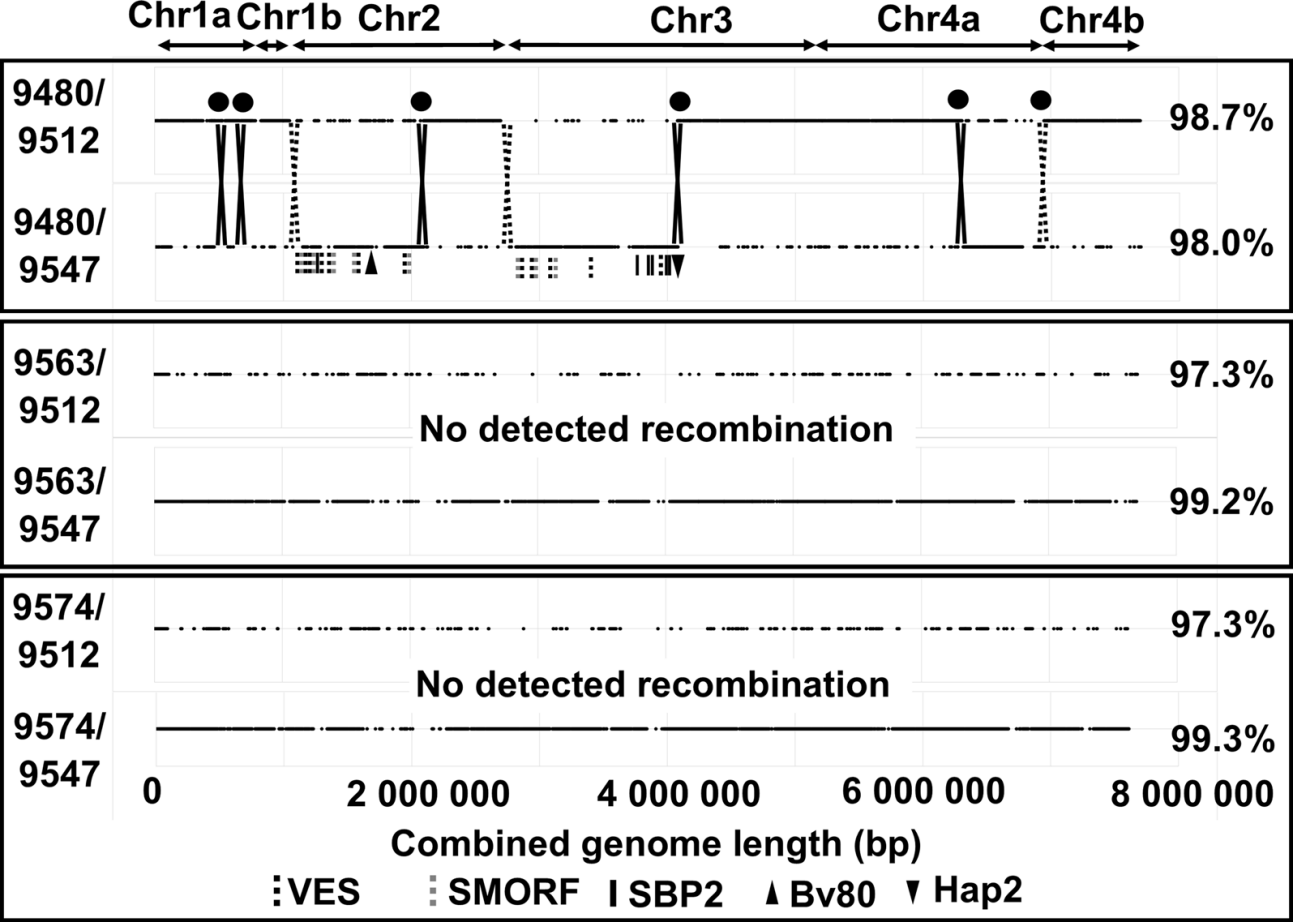
Potential recombination between S24 and 05-100. Indicated are the pairwise sequence comparisons (1 kb window across genome) of the S17.2-cl clones 9480-S24×05-100, 9563-S24×05-100 and 9574-S24×05-100 obtained from the co-transmission of S24 and 05-100 with 9512-S24 and 9547-05-100. Only regions of 100% identity are indicated by black lines. The average sequence identity is indicated on the right for each sequence pair and the corresponding regions for the major chromosomes are indicated on top. Crossover points due to chromosomes are indicated with dotted X’es while recombination events are indicated by solid X’es and the total recombination events by solid circles. Various genes are indicated by their respective symbols

Both S17.2-cl and S24 failed to infect ticks and transmit in a clonal genotype state. However, co-transmissibility was observed for both in the presence of other transmissible genotypes in this and previous studies [21]. A number of possible reasons were previously proposed for non-transmissibility/transmissibility and rescue during co-transmissibility [21]. One reason proposed was sexual recombination that allows tick transmittance factors to be acquired by non-transmissible genotypes. To assess the possibility that S24 and 05-100 sexually recombined before tick transmission, the genomes from 9512-S24, 9547-05-100 and the clones (9480-S24×05-100, 9563-S24×05-100, 9574-S24×05-100) previously obtained *via* limiting dilution [21], was compared. Pairwise sequence comparison of 9512-S24 and 9547-05-100 showed an average sequence identity of 97.2% across 7.1 Mbp, homogenously distributed throughout the genome (Fig. 3). It should therefore be possible to detect recombination events by pairwise comparison to identify regions that show 100% identity to either 9512-S24 or 9547-05-100 in the clones sequenced. Comparison of 9563-S24×05-100 and 9574-S24×05-100 with 9512-S24 and 9547-05-100 indicated average sequence identities of 97% and 99%, respectively (Fig. 5). This can also be observed in the random distribution of fragments with 100% identity compared to 9512-S24 that only cover a combined length of 1.1–1.2 Mbp, while comparison with 9547-05-100 gave a combined length of 5.4–5.8 Mbp visible as long continuous stretches (Fig. 5). Clones 9563-S24×05-100 and 9574-S24×05-100 are not considered to display any recombination signals with 9512-S24. Phylogenetic analysis do suggest that 9563-S24×05-100 and 9574-S24×05-100 may be divergent lineages from 05-100, or that they are S24×05-100 recombinants with very little recombination signal from S24, with the major parent being 05-100 (Fig. 4). Even so, 9547-05-100, 9563-S24×05-100 and 9574-S24×05-100 group within a well-supported clade suggesting a shared genetic relationship.

Conversely, comparison of 9480-S24×05-100 with 9512-S24 and 9547-05-100 indicate average sequence identities of 98.7% and 98.0%, respectively (Fig. 5). Fragments of 100% identity cover a combined length of 4.7 Mbp (66% combined length) and 3.0 Mbp (42% combined length) when compared to 9512-S24 and 9547-05-100, respectively. Visual comparison clearly shows alternating stretches of 100% identity between 9512-S24 and 9547-05-100 that covers a larger proportion of 9512-S24. Compared to the profiles obtained for 9563-S24×05-100 and 9574-S24×05-100, this suggests that 9480-S24×05-100 represent a recombination event between S24 and 05-100. Five major cross-over events may be identified that occurred on chromosome 1 (~0.075 Mbp for 9574-05-100; ~0.72 Mb for 9512-S24), chromosome 2 (~0.99 Mbp for 9547-05-100; ~0.61 Mbp for 9512-S24), chromosome 3 (~1.37 Mbp for 9547-05-100; ~1.1 Mbp for 9512-S24), chromosome 4a (~0.64 Mb for 9547-05-100; ~1.13 Mbp for 9512-S24) and an undefined locality that occurred between chromosome 4a and chromosome 4b resulting in chromosome 4b corresponding with 9512-S24 (~0.74 Mbp). These cross-over events were confirmed by manual inspection of genome sequence alignments of 9512-S24, 9547-05-100 and 9480-S24×05-100. Phylogenetic analyses also indicated that 9480-S24×05-100 group between the S24 and 05-100 clades, as may be expected if it shares similarity to both parent strains, namely S24 and 05-100 (Fig. 4).

### Variation within and between genomic datasets

**Fig. 6:**
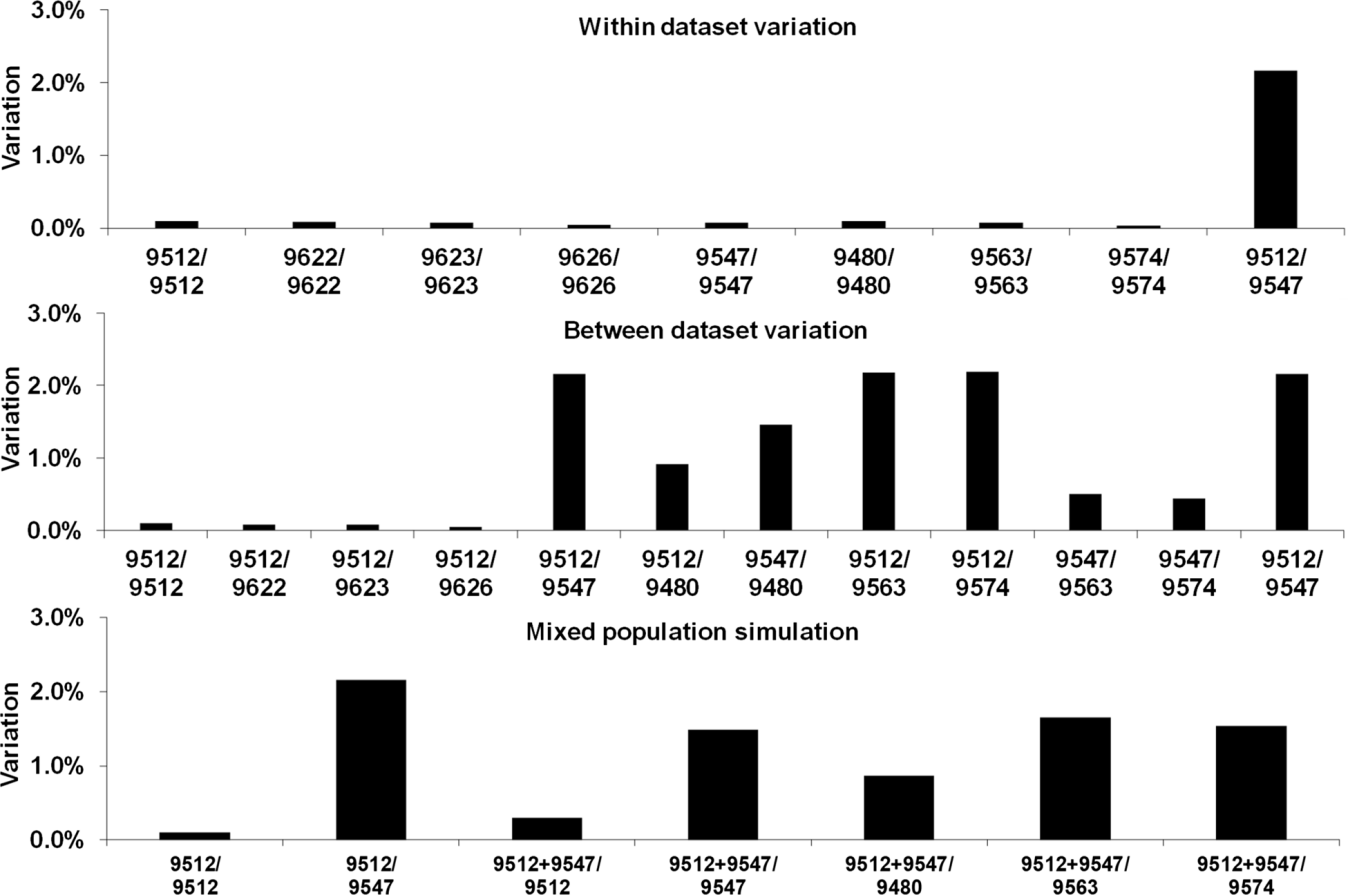
Assessment of variants within genomic datasets, between datasets and simulation of mixed populations within a dataset. Datasets (top animal number) were mapped to genomes of bottom animal numbers and variant calling was performed to assess genetic variation within the dataset. For simulation of mixed populations two datasets from 9512-S24 and 9547-05-100 were combined and mapped to various genomes

The question is raised whether the 9512-S24 vaccine strain, 9547-05-100 field strain and various clonal limiting dilution lines and the proposed recombinant clone 9480-S24×05-100 are indeed clonal lines and not contaminated with other genotypes. To address this, genetic variation was assessed by variant calling of each dataset to itself. This resulted in variation of ~0.1% or less within single datasets (Fig. 6). Those clonal lines (9622-17.2-cl, 9623-17.2-cl, 9626-17.2-cl) proposed to derive from the ancestral 9512-S24 population also showed less than 0.1% variation within their own datasets and compared to the 9512-S24 dataset (Fig. 6), supporting a common ancestry as suggested by phylogenetic analysis.

The clonal lines for which no recombination was detected (9563-S24×05-100 and 9574-S24×05-100), implying that they derived from recombination of 05-100 with itself, showed ~0.1% or less variation within their own datasets (Fig. 6). Even so, they do show higher variant percentages compared to 9547-05-100 as suggested by phylogenetic analysis as well, suggesting that while deriving from a common ancestral population, may be divergent from 05-100.

A simulation of mixed populations using combined datasets for 9512-S24 and 9547-05-100, mapped against the various assembled genomes resulted in much higher percentages of variation (0.3–2.2%) (Fig. 6). This is the signature expected for mixed populations and contrast to the similar levels of genetic diversity observed for all single datasets, suggesting that the genomes assembled do derive from clonal or dominant populations. The recombinant clone in the study, 9480-S24×05-100 shows levels of variation intermediate between single and simulation sets when compared to either 9512-S24 or 9547-05-100, suggesting again that it shared combined signals as may be expected for a recombinant clone.

## Discussion

Attenuation of live parasite cultures is a well-accepted approach to develop vaccines using whole organisms [15]. It generally entails repeated passage in live animals or cell culture to select for populations that are less virulent than the original strain. However, the attenuation process itself is not well understood and remain a stochastic process that introduce significant uncertainty into the process of vaccine production. Attenuation may be due to selection of less virulent populations, decrease in the genetic diversity of a parasite population or genetic changes within a population [18, 36]. It implies that attenuation is multifactorial and that attenuation of independent strains may not necessarily result in vaccines with similar genetic compositions. It also raises the interesting question whether attenuation of a specific virulent isolate is repeatable. In the present study this topic was investigated by revisiting the attenuation process of the South African S24 vaccine strain.

The S24 vaccine strain is characterized by a clonal population that exhibits the Bv80 A558 genotype [25]. Previously, this genotype was observed to appear between rapid syringe passage 16 and 18 of the original S11 vaccine strain, with a dramatic loss of the A645 and B585 genotypes. In an attempt to reconstruct this changeover in the vaccine composition, the original S16 frozen stock was used to generate S17.2 infection. This resulted in the appearance of the expected A558 genotype observed in the current S24 vaccine. It was also accompanied by a reduction in the signal for the A645 genotype. However, the B585 genotype was not reduced. Subsequent passage to generate a S18.2 strain did not result in further increase of the A558 genotype, but the passage reverted back to the genetic profile observed for the S16 passage, i.e. a strong signal for the A645 and B585. This suggests that while the appearance of the A558 genotype could be confirmed, the changeover in population structure could not be reproduced. It also suggests that creation of the current South African S24 vaccine strain was a serendipitous event, underscoring the genetic importance of this clonal line as National Asset, since recent studies confirmed its efficacy [37].

Part of the original protocol was the continuous passage of live parasites without any freezing steps between passages. The S17.2 strain from the present study was derived from a frozen S16 stock that has been maintained at −70 °C for 36 years. Whether this impacted on the inability of the A558 genotype to reestablish itself as dominant genotype is not known. It is considered that rapid passage may select for fast replicating populations resulting in loss of slow replicating populations. The absence of the tick transmitting genotype in some attenuated strains may support this, since these genotypes may lack certain genomic regions associated with tick transmission, resulting in smaller genome sizes or factors that impact on replication rate.

Remarkably, cloning by limiting dilution of the S17.2 strain resulted in the complete loss of both major genotypes (A645 and B585), with recovery of 50% of animals (*n* = 4) infected with the A558 genotype and 25% animals (*n* = 2) infected with a completely novel A535 genotype. This suggests that these genotypes may be prone to survival at very low parasitaemia, or less likely to be identified and removed by the splenectomized host’s immune system, or be sequestered in the host compared to the dominant genotypes. In this regard, the original storage of blood for 7 days at 4 °C prior to passage from S11 to S22 may have contributed towards the selection of the A558 genotype and loss of the A645 and B585 genotypes that resulted in lower numbers of parasites surviving between passages. Recovery of the A558 genotype by limiting dilution from S17.2, suggests alternative approaches to attain attenuation, by selection of defined clonal populations. This may be a viable approach if the current S24 vaccine strain should be lost by mishap.

Attempts to recover the S24 vaccine strain using limiting dilution previously failed on 34 attempts [21]. It is therefore quite surprising that the majority of genotypes recovered in the present study were for the A558 genotype. This may indicate that multiple A558 genotypes existed and that the S17.2 strains are different from the S24 vaccine strains. It may also suggest that the S24 A558 genotype underwent a significant genetic change during passage that may have included loss of tick transmissibility, loss of virulence and loss of ability to propagate after limiting dilution. In this regard, virulence and limiting dilution phenotypes have been linked before [38]. Given the high genetic similarity observed between the S24 and S17.2 genomes, it suggests that loss of virulence and limiting dilution phenotypes may be restricted to only few genes in the genome.

The present study again confirmed the non-transmissibility of the A558 genotype when present as a clonal population. Interestingly, its presence in the S17.2 strain did not result in significant co-transmission with the A645 and B585 genotypes. This may suggest that the levels of parasitaemia were not high enough to ensure co-transmission, which would correlate with its inability to appear in the S18.2 passage.

The present study attempted to reconstruct the events that led to replacement of the original dominant A645 and B585 genotypes found in the S16 passage, with the A558 genotype present in the S18 passage that was eventually used as the commercial vaccine at passage S24. While the A558 genotype was observed in passage S17.2, it failed to replace either A645 or B585 genotypes in the subsequent S18.2 passage. This suggests that passage from frozen stocks may not necessarily recapitulate historical passages.

The limiting dilution phenotype has previously been linked to cytoadherence and the ability to evade the host’s immune system [18, 38]. This has been linked to various potential genes, notably the variant erythrocyte surface antigen (VESA1) family, the small open reading frame (smORF) family and the spherical body protein (SBP2) family [38–41]. In the regions identified as potential recombined regions in 9480-S24×05-100 that derive from 05-100 there is 19 VES, 9 smORF and 2 SBP2 genes on chromosome 2 and 11 VES, 4 smORF and 11 SBP2 genes on chromosome 3 and none of these genes on chromosome 1 or chromosome 4a. These genes could potentially be involved in the limiting phenotype observed for 9480-S24×05-100.

The inability to obtain the A558 genotype during limiting dilution of S24 may suggest that the limiting dilution phenotype is genetically linked with the Bv80 gene (genetic proximity). Conversely, it would also suggest that the tick-transmissible and limiting dilution phenotypes are not genetically linked since the S16 vaccine strains that possessed the A558 allele exhibited the limiting dilution phenotype, although it could not be tick-transmitted. More work is however, required to confirm these possibilities. The regions identified as potential recombined regions in 9480-S24-05-100 that derive from 05-100 contain ~438 genes on chromosome 2, ~620 genes on chromosome 3 and ~290 genes on chromosome 4a. Of these, 68 are coding for potential membrane proteins that may act as receptors for gut invasion [31].

The molecular basis for tick transmission phenotypes has not been elucidated yet, given the various possibilities that exist which will result in a tick transmission or non-transmission phenotype. This may include inability to penetrate tick gut, salivary gland or ovary cells due to an absent/dysfunctional parasite receptor. Alternately, transmissible parasites secrete an enzyme in the tick gut that enables all parasites to penetrate the gut epithelium, even though absent in some genotypes [20]. Non-transmissible strains may acquire the enzyme during sexual recombination. The tick immune system may be able to kill non-transmissible strains [42–44]. In this case genes involved in tick immune responses would be absent from the non-transmissible strains. Tick-transmissible parasites may upregulate host proteins involved in parasite invasion [42]. Non-transmissible strains would lack the genes involved in host upregulation. In all of the above scenarios parasite genes are absent in non-transmissible strains and the A558 genotypes would have acquired the parasite receptor or protein involved in tick transmission through sexual recombination [19, 45]. Other possibilities may include differential gene expression in different parasite strains. Future experiments may include transcriptome analysis to determine whether gene expression differences could be linked with the non-transmissible phenotype.

Another possibility would be that genes involved in development of the sexual stage in the tick are responsible for the non-transmissible phenotype of S24. These may include the 6-Cys gene superfamily (A-J) or the hap2 gene [46, 47]. This, however, seems a remote possibility since the 6-Cys genes are intact in all sequenced genomes and in recombinant 9480-S24×05-100 all derive from the S24 strain. In recombinant 9480-S24-05-100, hap2 derive from 05-100 and may contribute to the tick transmissibility phenotype in recombinant 9480-S24-05-100. However, hap2 are also intact in all genomes sequenced including 9512-S24 suggesting that it would be functional in the S24 strain. Given that 9480-S24×05-100 derive partly from S24, it suggests that the S24 vaccine strain is capable of sexual recombination.

Sexual recombination has been accepted as part of the life-cycle of *Babesia* parasites [48]. Development of blood stage merozoites to gametocytes, division to gametes after tick ingestion, sexual recombination by haploid gametes to form diploid zygotes that infect the gut as ookinetes, with subsequent division into haploid kinetes inside the gut epithelium [48], has been well documented using light and electron microscopy [49, 50]. The haploid nature of merozoites and gametes and the diploid nature of the zygote has been demonstrated using flow cytometry [51], indicating sexual fusion. This seems to be a general feature of all protists [52]. However, to date no genetic data have confirmed sexual recombination in *B. bovis*, although sexual recombination could be expected given its occurrence in other Piroplasmida such as *Theileria* [53]. The present study confirmed sexual recombination in *B. bovis* using genetic data that show a clonal line obtained after co-transmission sharing significant stretches of identity with either parental strain.

A low cross-over frequency was found compared to other protozoan parasites such as *Plasmodium falciparum* [54] and *Theileria parva* [55]. However, the number satisfies the obligatory number of crossover events necessary for successful meiosis and falls well within the average of 1–2 crossover events per chromosome observed for many organisms [56]. The low number may be explained by the clonal population that was analyzed and would reflect the recombination history of a single clone, rather than that of a population. However, the purpose of the present study was not the fine mapping of recombination, but to identify in a broad sense possible genomic regions that may be involved with the tick transmissible and limiting dilution phenotypes.

## Conclusions

The present study investigated the development of the current S24 vaccine and showed that its selection during rapid passage was a serendipitous event. The resulting vaccine showed both non-transmissible and lack of limiting dilution phenotypes, the latter possibly responsible for its attenuation. It confirmed its inability to be tick transmitted and showed that sexual recombination is probably responsible for tick co-transmission. To our knowledge, this is also the first genetic evidence of sexual recombination for *Babesia bovis*. Progressive crossing of 05-100 recombinant lineages with the S24 vaccine stock, using the tick transmission and limiting dilution phenotypes as selection criteria may result in identification of the genes responsible for both genotypes. These genes may be potential candidates for development of transmission blocking or sequestration blocking vaccines or dual blocking vaccines.

## Supplementary information

**Additional file 1: Table S1** Summary of the *de novo* assembly of the *Babesia bovis* genome compared to the reference genome size for the chromosomes and organelles. The size of the reference genome is indicated and the relative size of each *de novo* assembled genome in percentage. Highlighted chromosomes were used for the analysis of recombination and genome similarity.

**Additional file 2: Table S2.** Mapping statistics for reads to their respective *de novo* assembled genomes. Indicated are the number of paired-end reads generated after quality trimming, the number mapped to their respective *de novo* assembled genomes, the number of reads mapped in pairs; and the percentage reads mapped and the percentage reads mapped in pairs.

**Additional file 3: Table S3.** Summary of the mapping coverages obtained for the major chromosomes *de novo* assembled for various *B. bovis* clones.

## Data Availability

Sequence data supporting the findings of this study have been deposited in public sequence databases. Raw sequence reads have been deposited in the NCBI Short Read Archive (SRA, SRR9678899– SRR9678960) under Bioproject accession number PRJNA552727 and are available from http://www.ncbi.nlm.nih.gov/Traces/sra/.

## Abbreviations

Bv80: *Babesia bovis* gene Bv80
S24: South African S24 vaccine strain obtained after 23 rapid needle passages of the S strain
05-100: field strain obtained from bovine exhibiting clinical *Babesia* symptoms
9512-S24: S24 vaccine strain grown in bovine 9512
9547-05-100: field strain 05-100 grown in bovine 9547
9522-S17.2-cl: clone obtained by limiting dilution from the S17.2 vaccine strain
9523-S17.2-cl: clone obtained by limiting dilution from the S17.2 vaccine strain
9526-S17.2-cl: clone obtained by limiting dilution from the S17.2 vaccine strain
9480-S24×05-100: clone obtained by limiting dilution that derived from co-transmission of S24 and 05-100 grown in bovine 9480
9563-S24×05-100: clone obtained by limiting dilution that derived from co-transmission of S24 and 05-100 grown in bovine 9563
9574-S24×05-100: clone obtained by limiting dilution that derived from co-transmission of S24 and 05-100 grown in bovine 9574
