# Supplementary material for: Investigation into limiting dilution and tick transmissibility phenotypes associated with attenuation of the S24 vaccine strain
Source: Parasit Vectors. 2019 Aug 27;12:419. doi: 10.1186/s13071-019-3678-2 (PMC6712794; doi:10.1186/s13071-019-3678-2)
Supplement: Supplementary file 1 — Additional file 1: Table S1 Summary of the de novo assembly of the Babesia bovis genome compared to the reference genome size for the chromosomes and organelles. The size of the reference genome is indicated and the relative size of each de novo assembled genome in percentage. Highlighted chromosomes were used for the analysis of recombination and genome similarity. [file 13071_2019_3678_MOESM1_ESM.docx]

**Additional file 1: Table S1.** Summary of the *de novo* assembly of the *Babesia bovis* genome compared to the reference genome size for the chromosomes and organelles. The size of the reference genome is indicated and the relative size of each *de novo* assembled genome in percentage.

|  | **Reference genome**  **(Size bp)** | **9512**  **(S24)** | **9547**  **(05-100)** | **9563**  **(S24x05-100)** | **9480**  **(S24x05-100)** | **9574**  **(S24x05-100)** | **9622**  **(S17.2cl)** | **9623**  **(S17.2cl)** | **9626**  **(S17.2cl)** |
| --- | --- | --- | --- | --- | --- | --- | --- | --- | --- |
| **Apicoplast** | 35,107 | 79.6% | 98.1% | 84.83208 | 90.8% | 50.6% | 61.0% | 84.8% | 26.2% |
| **^a^Chr1a** | 821,816 | 83.8% | 91.0% | 90.6% | 93.8% | 90.0% | 93.4% | 91.7% | 86.0% |
| **^a^Chr1b** | 285,379 | 90.7% | 83.8% | 85.9% | 90.1% | 83.1% | 90.6% | 91.9% | 82.6% |
| **Chr1c** | 28,266 | 39.5% | 24.8% | 30.4% | 44.9% | 20.3% | 45.3% | 45.4% | 23.4% |
| **Chr1d** | 15,708 | 79.2% | 34.1% | 52.4% | 55.0% | 44.1% | 63.3% | 68.1% | 41.6% |
| **Chr1e** | 13,770 | 66.0% | 23.2% | 45.2% | 50.2% | 28.0% | 54.2% | 71.5% | 28.5% |
| **Chr1f** | 12,362 | 66.7% | 26.3% | 46.0% | 66.6% | 16.2% | 69.1% | 72.5% | 23.6% |
| **Chr1g** | 11,916 | 49.9% | 27.6% | 48.6% | 32.7% | 36.2% | 42.1% | 58.5% | 30.5% |
| **^a^Chr2** | 1,729,419 | 94.5% | 95.7% | 94.5% | 92.7% | 93.1% | 94.9% | 93.89% | 90.6% |
| **^a^Chr3** | 2,593,320 | 95.2% | 95.7% | 94.7% | 95.3% | 93.7% | 95.9% | 94.0% | 90.6% |
| **^a^Chr4a** | 1,797,577 | 97.7% | 97.4% | 64.0% | 98.3% | 96.5% | 96.2% | 94.0% | 90.2% |
| **^a^Chr4b** | 827,912 | 90.1% | 96.4% | 96.3% | 90.3% | 97.7% | 97.1% | 91.6% | 88.5% |
| **Chr4c** | 1,149 | 0 | 0 | 0 | 0 | 0 | 0 | 0 | 0 |
| **Mitochondrial genome** | 6,005 | 98.5% | 98.5% | 98.5% | 98.5% | 98.5% | 98.5% | 98.5% | 98.5% |

^a^Chromosomes were used for the analysis of recombination and genome similarity
