# Supplementary material for: Investigation into limiting dilution and tick transmissibility phenotypes associated with attenuation of the S24 vaccine strain
Source: Parasit Vectors. 2019 Aug 27;12:419. doi: 10.1186/s13071-019-3678-2 (PMC6712794; doi:10.1186/s13071-019-3678-2)
Supplement: Supplementary file 2 — Additional file 2: Table S2. Mapping statistics for reads to their respective de novo assembled genomes. Indicated are the number of paired-end reads generated after quality trimming, the number mapped to their respective de novo assembled genomes, the number of reads mapped in pairs; and the percentage reads mapped and the percentage reads mapped in pairs. [file 13071_2019_3678_MOESM2_ESM.docx]

**Additional file 2: Table S2.** Mapping statistics for reads to their respective *de novo* assembled genomes. Indicated are the number of paired-end reads generated after quality trimming, the number mapped to their respective *de novo* assembled genomes, the number of reads mapped in pairs; and the percentage reads mapped and the percentage reads mapped in pairs.

|  | **9512**  **(S24)** | **9547**  **(05-100)** | **9480 (S24x05-100)** | **9563**  **(S24x05-100)** | **9574**  **(S24x05-100)** | **9622**  **(S17.2cl)** | **9623 (S17.2cl)** | **9626**  **(S17.2cl)** |
| --- | --- | --- | --- | --- | --- | --- | --- | --- |
| **Number of paired-end reads** | 8,569,522 | 12,339,174 | 8,937,818 | 3,109,714 | 3,267,736 | 7,577,280 | 6,966,434 | 4,454,710 |
| **Number of mapped reads** | 8,125,977 | 5,781,719 | 8,517,255 | 1,563,453 | 3,131,094 | 7,367,106 | 6,748,346 | 4,186,009 |
| **Number of reads mapped in pairs** | 7,145,940 | 5,427,760 | 7,597,690 | 1,502,290 | 2,757,610 | 6,939,346 | 6,510,878 | 3,954,738 |
| **Percentage mapped** | 94.8% | 46.8% | 95.3% | 50.3% | 95.8% | 97.2% | 96.9% | 93.9% |
| **Percentage mapped in pairs** | 83.3% | 43.9% | 85.0% | 48.3% | 84.4% | 91.6% | 93.5% | 88.8% |
