# Supplementary material for: Investigation into limiting dilution and tick transmissibility phenotypes associated with attenuation of the S24 vaccine strain
Source: Parasit Vectors. 2019 Aug 27;12:419. doi: 10.1186/s13071-019-3678-2 (PMC6712794; doi:10.1186/s13071-019-3678-2)
Supplement: Supplementary file 3 — Additional file 3: Table S3. Summary of the mapping coverages obtained for the major chromosomes de novo assembled for various B. bovis clones. [file 13071_2019_3678_MOESM3_ESM.docx]

**Additional file 3: Table S3.** Summary of the mapping coverages obtained for the major chromosomes *de novo* assembled for various *Babesia bovis* clones.

|  | **9512**  **(S24)** | **9547**  **(05-100)** | **9480**  **(S24x05-100)** | **9563**  **(S24x05-100)** | **9574**  **(S24x05-100)** | **9622**  **(S17.2cl)** | **9623**  **(S17.2cl)** | **9626**  **(S17.2cl)** |
| --- | --- | --- | --- | --- | --- | --- | --- | --- |
| **Chr1a** | 164.8 | 113.3 | 165.8 | 30.2 | 60.7 | 197.7 | 95.3 | 65.4 |
| **Chr1b** | 166.9 | 119.2 | 169.8 | 30.8 | 60.7 | 102.4 | 100.9 | 71.3 |
| **Chr2** | 174.7 | 119.5 | 171.1 | 32 | 63.8 | 106.4 | 98.9 | 67.2 |
| **Chr3** | 170.1 | 116.6 | 167.8 | 30.7 | 62.2 | 104.4 | 97 | 65.3 |
| **Chr4a** | 164.5 | 110.6 | 161.9 | 28.8 | 56.6 | 96.3 | 89.3 | 59.2 |
| **Chr4b** | 172.3 | 118.8 | 172.9 | 30.5 | 65 | 106.9 | 99.7 | 65.7 |
| **Average** | 168.9 | 116.3 | 168.2 | 30.5 | 61.5 | 119.0 | 96.9 | 65.7 |
